# Contemporary nutrition-based interventions to reduce risk of infection among elderly long-term care residents: A scoping review

**DOI:** 10.1371/journal.pone.0272513

**Published:** 2022-08-02

**Authors:** Athanasios Psihogios, Claudia Madampage, Brent E. Faught

**Affiliations:** 1 Department of Health Sciences, Faculty of Applied Health Sciences, Brock University, St. Catharines, ON, Canada; 2 School of Public Health, University of Saskatchewan, Saskatoon, Canada; Western Sydney University and University of New South Wales, AUSTRALIA

## Abstract

**Background:**

Elderly long-term care residents (ELTCRs) face considerable burden of infection, especially evident during the COVID-19 pandemic. The nutritional status of the host can influence susceptibility to infection by altering immune system integrity, therefore, nutrition-based interventions may be a viable complement to existing infection prevention measures.

**Objective:**

This scoping review sought to identify nutritional interventions and factors that have the strongest evidence to benefit ELTCRs, and thus best poised for rigorous clinical trial evaluation and subsequent implementation.

**Methods:**

A database search of OVID-Medline, OVID-Embase, and Web of Science was performed from 2011 to 2021 to identify nutritional intervention studies which attribute to changes in infection in contemporary ELTCR settings. Articles were screened in duplicate and data extraction completed by a single reviewer, while a second reviewer verified the data which was fitted to identify evidence for nutritional interventions related to reducing rates of infection among ELTCRs.

**Results:**

The search identified 1018 studies, of which 11 (nine clinical trials and two observational cohort studies) satisfied screening criteria. Interventions that significantly reduced risk of infection included whey protein (any infection), Black Chokeberry (urinary tract infection), and vitamin D (acute respiratory tract infection, skin and soft tissue infection). Both zinc and a dedicated meal-plan significantly improved lymphocyte parameters. Vitamin D deficiency was associated with the development of respiratory tract infections. Probiotic and soy-based protein interventions did not significantly affect risk of infection or lymphocyte parameters, respectively.

**Conclusion:**

The current scoping review was effective in identifying the use of nutrition-based interventions for infection prevention among ELTCRs. In this study, some nutrition-based interventions were observed to significantly influence the risk of infection among ELTCRs. Nutritional interventions such as vitamin D (preventing deficiency/insufficiency), Black Chokeberry juice, zinc gluconate, whey protein, and varied and nutrient dense meal plans may be suitable for future rigorous clinical trial evaluation.

## Introduction

Elderly long-term care residents (ELTCRs) face a considerable burden of infection, including high rates of respiratory tract infections [1], urinary tract infections [2], sepsis [3], and more recently, a disproportionate vulnerability to severe manifestations of acute respiratory syndrome coronavirus 2 (SARS-CoV-2) [4–7]. Risk factors for infection among ELTCRs include the presence of numerous comorbidities, functional decline and/or disability, cognitive impairment, dental disease resulting in loss of teeth, use of medical devices to support health-related conditions, and malnutrition [8]. While no internationally accepted standard diagnostic method for identifying malnutrition exists, the assessment often involves a multi-step approach involving different validated tools [9]. Even though some variations exist, most malnutrition definitions and assessments include components of weight loss, BMI measures, and poor food intake [10]. Relative to other risk factors, malnutrition is possibly a viable modifiable option for infection control among ELTCRs [11, 12], as nutritional status directly influences a host’s susceptibility to infection [13–15], and more importantly, can be altered through simple means (e.g., supplementation with vitamins, evaluation of dietary needs, and improving access to nutritional food).

Among ELTCRs, age-associated waning of systemic immunity (known as immunosenescense) may contribute towards increased risk and severity to infectious diseases [16]. An optimally functioning immune system is dependent on both age and adequate and appropriate nutrition. The immune system may progress towards reducing host protection beginning at the sixth decade of life due to decreased microbial immunity, predisposition for atherosclerotic disease, osteoarthritis or many neurodegenerative diseases, and decreased responses to protect against malignancies and wound healing [17]. To predict risk and severity to infectious diseases, the use of immune biomarkers is useful and will further provide opportunities for clinical intervention [18]. Changes in T-cells have been associated with immune biomarkers such as reduction in the number of naïve T-cells, an increase in CD8+ memory T-cells subsets, and an increase in senescent cells (CD8+ CD28-) [18]. Older adults may require additional nutrition due to energy demands, which are further increased during periods of infection necessary for robust immune responses [14]. Nutrient-specific immune system functions have also been identified, such as the need for adequate vitamin A and zinc for effective proliferative immune responses [14], vitamin E for maintaining and enhancing natural killer (NK) cell cytotoxic activity [19], and the interaction between vitamin D and its receptors present on most immune cells that broadly effect both the innate and adaptive systems [15].

In practice, malnutrition, as determined by validated nutritional assessment tools, has been observed to increase the risk of serious infections (lower respiratory tract infection, healthcare-associated infections, and a weakened immune system) [20–22], urinary tract infections [23], and pneumonia [24] among malnourished ELTCRs compared to their counterparts who are adequately nourished. Furthermore, specific nutrient deficiencies have also been implicated with infection in this population. The deficiency of zinc is associated with both incidence and duration of pneumonia [25] and vitamin D deficiency with the risk of developing respiratory tract infections [26].

These observations are relevant to Canadian ELTCR cohorts, as nutrient intake deficits and/or malnutrition is reported to be widespread and persistent in long-term care facilities across the nation. A 2018 Canadian cross-sectional study conducted in thirty-two long-term care homes (n = 632 randomly selected residents) found that >50% of residents consumed inadequate amounts of vitamin D, E, K, B6, folate, calcium, and magnesium [27]. Another publication by the same authors using the same patient cohort, found 44% of residents were also considered to be malnourished [28]. A similar study in 2008, investigated inadequacies in nutrient intake among five elderly long-term care institutions in Saskatoon (n = 48). The study reported a substantial proportion of residents consumed inadequate folate, magnesium, zinc, vitamin C, E, B6, protein, and thiamine [29]. Calcium, vitamin D, and dietary fibre were also substantially below the recommended intake [29]. A Toronto based cross-sectional study of 11 long-term care facilities (n = 407 residents) found that 30% of residents had low protein intake, >50% had suboptimal calcium, magnesium, zinc, vitamin E, B6, and folate intake, and 15% missed vitamin C, niacin and copper targets [30]. Finally, a small pilot study from Western Canada, assessing the overall nutritional status of veterans living in long-term care facilities (n = 55 residents), found that 58% were considered at risk for malnutrition, while 31% were already malnourished [31].

To our knowledge, no scoping review encompassing evidence for nutritional interventions in the realm of reducing rates of infection among ELTCRs has been published. A scoping review would facilitate the delineation of data on the effects of nutrition-based interventions to reduce the risk of infection among ELTCRs and facilitate in identifying areas of research to inform future experimental studies and pragmatic approaches to reduce the burden of infection in long-term care facilities. This present scoping review, through a detailed and structured literature search of peer reviewed articles, will attempt to describe the current evidence for the use of nutrition-based interventions to address infections in ELTCRs. The present scoping review is used to identify nutritional interventions and factors that have the strongest evidence to benefit this cohort, and thus, are best suited for rigorous clinical trial evaluation and subsequent implementation.

### Objectives of this study

1. To methodically map the extent, range, and nature of published, peer-reviewed, research related to nutrition-based interventions to reduce the risk of infection among ELTCRs [32, 33].
2. To identify nutrition-based interventions that have demonstrated the capacity to significantly reduce the risk of infection and/or improve clinically relevant immune system parameters (e.g., lymphocyte count) among ELTCRs, which may be suitable for future rigorous clinical trial evaluation.
3. To identify nutrition related parameters, evaluated in prospective cohort studies, demonstrating an association between undernutrition and/or nutrient deficiencies, and the risk of infection among ELTCRs.

## Materials and methods

The process of a scoping review facilitates identifying existing peer-reviewed literature that provides information that has not been comprehensively reviewed or is complex and diverse in nature [34]. A scoping review of recent academic literature on the current evidence of nutrition-based interventions to address infections in ELTCRs was conducted. Institutional research ethics approval was not required from Brock University as data was obtained from published literature in the public domain. This study was not registered with International Prospective Register of Systematic Reviews (PROSPERO: https://www.crd.york.ac.uk/prospero/) as it is not a systematic review.

### Methodological approach

The scoping review was conducted using a structured approach presented and informed using guidelines for a preferred reporting of items for systematic reviews and meta-analyses (PRISMA) extension for Scoping Reviews (PRSIMA-ScR) as shown in (S1 Table) [32]. A standard approach was applied to map the literature landscape related to the select topic through a process of: (i) identifying relevant studies; (ii) selection of studies; (iii) data extraction; and (iv) summarisation of results [33, 35].

### Eligibility criteria

#### Studies meeting the following inclusion criteria were included

- Published between 2011–2021.
- Full-text accessible for review.
- Published in English (to meet the language capabilities of all investigators).
- Peer-reviewed published literature.
- Either experimental/interventional (single-arm trial, randomized controlled trial (RCT), phase I-II clinical trials) or longitudinal epidemiologic study.
- Included a primary intervention that was nutrition based (including dietary supplements, meal plans, special foods, eating schedules, fortified drinks, etc.) or a mixed intervention that included nutritional reporting.
- At least one outcome or measure was infection related, including risk of infection (any type of infection), immune system parameters [T-cell (a type of immune cell), natural killer (NK) cells, etc.], the need for antibiotic use for present infection or other opportunistic infections, length of hospital stay due to infection, other complications due to infection, recovery, and response to vaccination.
- Participants were elderly (≥ 65 years of age). Studies with mixed aged groups were allowed, if the majority (>50%) were ≥ 65 years. The cut off for age (≥ 65 years) was based on the conventional definition of an ‘elderly’ individual to be defined as a chronological age of 65 years [36].
- Participants resided in any type of long-term care facility, such as nursing homes, throughout the intervention period.

#### Studies which met the following exclusion criteria were excluded

- The absence of a peer-review process.
- Unpublished results or literature.
- The following study designs: non-longitudinal observational studies, case reports, review, animal/pre-clinical/*in vitro studies*, conference abstracts/presentations/posters, editorials, and opinions.
- Did not include nutritional related primary intervention/s.
- Included participants that were primarily of community dwelling, hospitalised, with an acute care setting, in transitional centres, and/or were not within the guidelines of living in long-term care facility.
- Did not include outcome(s) or measure(s) related to infection.

### Sources of information

Three databases were searched for published, peer-reviewed, literature, including Web of Science (Core Collection) on May 31^st^, 2021, and both Ovid-Medline and Ovid-Embase on June 7^th^, 2021. Grey literature was not included as the intention of this scoping review was to specifically identify possible nutrition-based intervention candidates that can be evaluated and tested in future rigorous clinical trials. Thus, a peer-reviewed process for publication was essential and hence required. The primary reason for choosing 10 years (2011 to 2021) for all three databases was to ensure the collection would most closely reflect contemporary ELTCRs in the population, and thus would be most applicable and relevant to modern and future real-world cohorts. Nevertheless, an initial search with a 20 to 25-year time span (2001–2021 & 1996–2021) with the above-mentioned inclusion and exclusion criteria was implemented. This increased time frame included a very large number of citations. Following removal of duplicates, the remaining studies were not appropriate for nutrition-based interventions. Therefore, the rationale for a 10-year time span was adopted to reflect contemporary nutrition-based interventions among this cohort. Further, there is evidence of updates and revised guidelines pertaining to infection control for long-term and health care institutions in Canada [37–41], and internationally [42–44], that have occurred within the past 10 years, indicating an evolving approach. Thus, we included studies that would be most applicable for a contemporary setting.

### Database search terms

Search terms and filters with punctuation and conventions were used to provide a comprehensive landscape of all literature. The use of an asterisk (*) broadened a search by identifying words that begin with the same letters, while the use of combinations of free text keywords, acknowledged as many appropriate records as possible within a search, compared to a single keyword. Conventions such as: (i) exp = “explodes”, controlled vocabulary terms used to expand a search related to specific terms within the vocabulary hierarchy, and (ii) mp. = combined search fields, were incorporated when searching key words in OVID (online bibliographic databases) syntax [45]. The use of three Boolean operators (“AND”, “OR”, and “NOT”) provided a process to combine, narrow down (concentrate), refine, broaden or exclude keywords within the database search. In another vein, Boolean operator “OR” was used in parallel as a filter for key terms searched with the convention of medical subject heading (MeSH) [46]. Finally, after all key search terms were incorporated, Boolean operator “AND” was used to combine these sets [46]. All three databases were searched using search terms: “long-term care resident*”, “nursing care home*”, “nursing home*”, “home for the age*”, “elder*”, “old people*”, “age*”, “infect*”, “sick*”, “ill*”, “clinical nutrition”, “nutrition”, “dietary supplement*”, “vitamin*”, “mineral*”, “nourishment”, “diet*”, “meal*”, and “supplementation”. MeSH terms were then searched, with relevance to both Ovid-Medline and Ovid-Embase databases (Web of Science does not support the use of MeSH terms), including “long-term care”, “aged”, “home(s) for the aged”, “infection”, and “nutrition”. The unique MeSH term “nutrition therapy” was accessible and applied in Ovid-Medline while “geriatric nutrition” was available and applied in Ovid-Embase. All three databases facilitated filters to be applied regarding human population studies.

### Selection process and filtering data

The collection of retrieved articles from each individual database were exported to Zotero and duplicate results were removed using appropriate software functions. All deduped records were then uploaded to Rayyan, a free online web based tool used for screening articles encompassing systematic reviews, scoping reviews or environmental scans [47]. All titles, abstracts, and subsequently full texts were reviewed and screened for inclusion and exclusion criteria by the primary investigator (AP) and verified by a second co-investigator (CM). The final list of included studies was reviewed through chain searching to identify additional eligible studies not captured in the original database search. Chain searching, also known as backward reference searching, provides a process to examine studies stated within an article and reference it if applicable [48].

### Data charting

Once all eligible full text publications were identified, data extraction was performed by the primary investigator (AP) and verified by a second co-investigator (CM) to identify and organise evidence for nutritional interventions related to reducing rates of infection among ELTCRs. A data extraction form was created with the following variables [identified from each study include]: year of publication; study design; data collection period; sample size and characteristics; type of long-term care institution; country of origin; nutrition components details; infection related outcomes; infection related results; study limitations and strengths; and keywords related to the publication.

### Annotated bibliography

An annotated bibliography was developed (S1 File) with the following criteria applied to the 11 articles included in this scoping review: summary of source (author, purpose, relevance to our topic, accuracy); the strengths and limitations of the study; affiliation to the purpose of our work and study conclusion(s). The annotated bibliography is intended to provide a brief description or an evaluative paragraph about each article used for data extraction. No formal critical appraisal was conducted as it is beyond the scope of this type of work (a non-systematic, scoping review).

### Synthesis of results

Extracted data was organised into tables to present general characteristics of each included study. Results were organised by using the type of nutritional intervention relative to infection related outcomes.

## Results

### Results from database search

**Fig 1:**
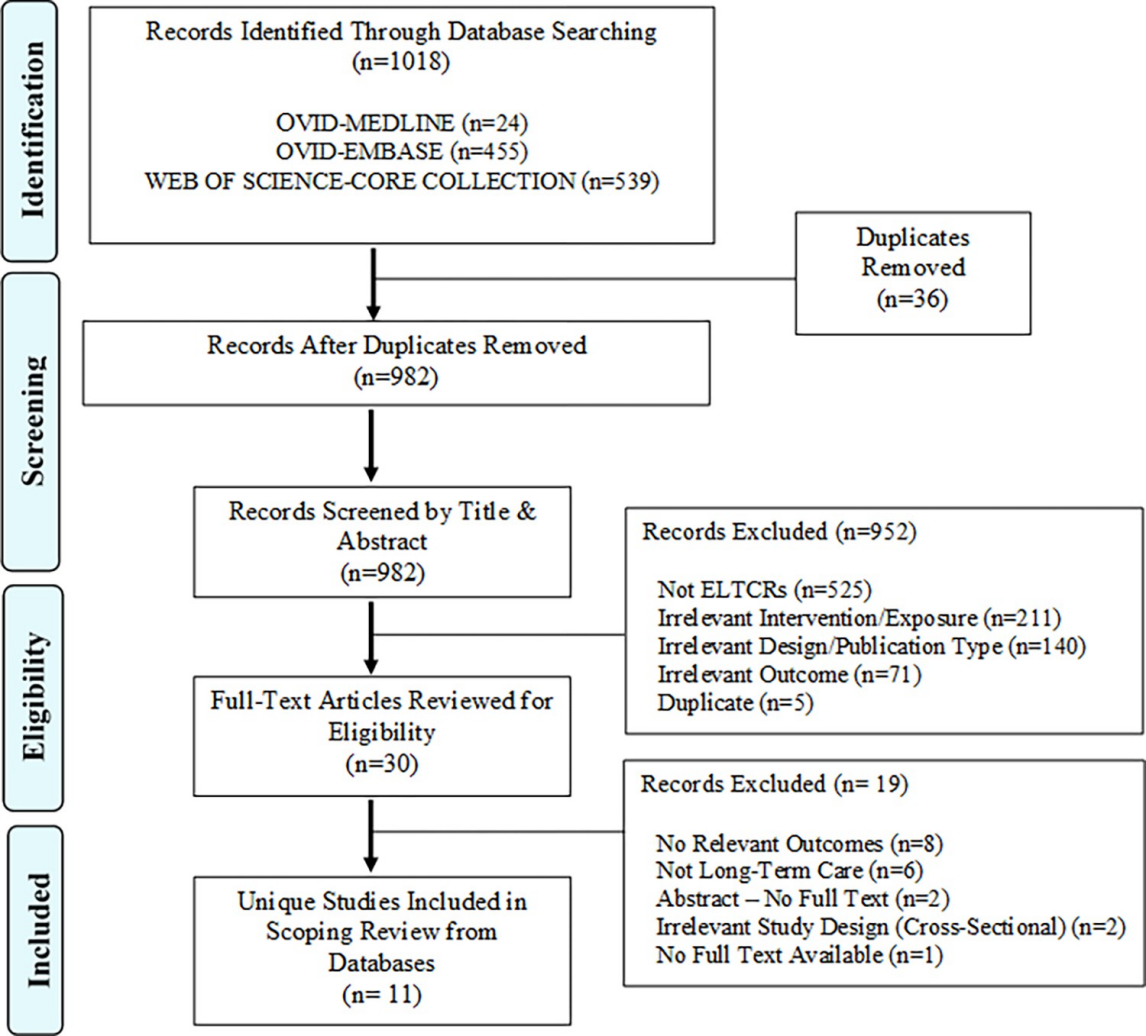
PRISMA flow chart.

Database searches of Ovid-Medline, Ovid-Embase, and Web of Science identified 1018 articles (after de-duplication). Following inter-database de-duplication (n = 36 articles removed), 982 records were screened by title and abstract for inclusion, resulting in 952 articles being excluded. The remaining 30 records were screened via full text, resulting in 19 exclusions, yielding 11 articles for data extraction. Chain searching of the 30 articles recognized for full text review resulted in no additional eligible studies being identified. Detailed depiction of study identification, screening, eligibility assessment, and inclusion is presented as a PRISMA flow chart (Fig 1).

## Characteristics of included studies

All 11 articles selected for data extraction were conducted in high income countries with ten having membership with the organisation for economic co-operation and development (OECD) and one a non-OECD member. Nine of the identified studies were experimental [49–57] and two observational [26, 58] (Table 1). Of the nine experimental, four were randomized, double-blind, placebo controlled clinical trials [50, 51, 54, 55], one was a randomized, double-blind, controlled clinical trial without placebo [56], one was a randomized, un-blinded, controlled clinical trial [52], one was a group-randomized, double-blind, placebo controlled, crossover clinical trial [53], one was a pragmatic participatory evaluation trial [49], and one was an uncontrolled, pre-post, single-arm clinical trial [57]. Both observational studies applied a prospective cohort design [26, 58]. All study settings were in nursing homes, except for one that recruited residents from long-term care facilities with a mix of skilled nursing homes and assisted living facilities [54]. Intervention administered in the experimental studies included probiotics (n = 3) [49–51], a protein-based product (n = 2) [52, 56], Black Chokeberry (*Aronia melanocarpa*) juice (n = 1) [53], dedicated, needs-specific, meal plan (n = 1) [57], high dose vitamin D (n = 1) [54], and zinc gluconate n = 1) [55].

**Table 1:** Characteristics of included studies.

Study Design	Study Details	Nutritional-based Intervention	Study location	Sample size and baseline characteristics of study participants
Experimental study design (n = 4)	Randomized, double-blind, placebo controlled clinical trials	Probiotics	Belgium [50]	N = 737 [50]
				**Placebo Arm:** Mean age 84.17 (range: 55–101); 277 females, 85 males
				**Probiotic Arm:** Mean age 83.95 (range: 64–101); 276 females, 99 males
		Probiotics	Canada [51]	N = 209 [51]
				**Placebo Arm:** Mean age 85.9 (+/- 7.0); 58 females, 38 males
				**Probiotic Arm:** Mean age 85.2 9+/- 7.1); 74 females, 26 males
		High dose vitamin D	USA [54]	N = 107 [54]
				**Standard Dose Arm:** Mean age 82 (+/- 10.0); 29 females, 23 males
				**High Dose Arm:** Mean age 80 (+/- 10); 33 females, 22 males
		Zinc gluconate supplement	USA [55]	N = 31 [55]
				**Placebo Arm:** Mean age 84.4 (+/-8.8); 12 females, 4 males
				**Zinc Arm:** Mean age 87.0 (+/- 5.0); 11 females, 4 males
Experimental study design (n = 1)	Randomized, double-blind, controlled clinical trial without placebo	Protein-based product (soy)	Taiwan [56]	N = 92 [56]
				**Control Group:** Mean age 80.2 (+/-7.8); 25 females, 20 males
				**Intervention Group:** Mean age 78.9 (+/-8.4); 28 females, 19 males
Experimental study design (n = 1)	Randomized, un-blinded, controlled clinical trial	Protein-based product (whey)	Finland [52]	N = 106 [52]
				**Control Group:** Mean age 83.0 (+/-8.7); 43 females, 14 males
				**Intervention Group:** Mean age 84.1 (+/- 7.6); 37 females, 12 males
Experimental study design (n = 1)	Group-randomized, double-blind, placebo controlled, crossover clinical trial	Black Chokeberry Juice*	Norway [53]	N = 236 [53]
				Mean age 85***; 160 females 76 males
Experimental study design (n = 1)	Pragmatic participatory evaluation trial**	Probiotics	Netherland [49]	N = 93 [49]
				**No Probiotics Group:** Mean age 83 (+/- 10.7); 35 females 14 males
				**Probiotics Group:** Mean age 85 (+/- 9.6); 48 females, 22 males
Experimental study design (n = 1)	Uncontrolled, pre-post, single-arm clinical trial	Needs-specific meal plan	Italy [57]	N = 479 [57]
				Mean age 79.72 (+/-12.31) (88.28% ≥65); 334 females, 67 males
Observational study design (n = 1)	Prospective cohort study		Japan [26]	N = 148 [26]
				Median age 87.0 (79.0–91.0); 117 females, 31 males
Observational study design (n = 1)	Prospective cohort study		Germany [58]	N = 200 [58]
				Mean age 85.5 (+/- 7.8); 147 females, 53 males

* Black Chokeberry is a species in the rose family with its fruit containing high amounts of vitamin C and polyphenolic compounds (which exert antioxidant properties) [59].

** Participatory action-based research which adapts a pragmatic design includes active engagement of healthcare workers to support outcomes suitable for nursing home practice [49].

*** The range or standard deviation is not reported.

USA: United States of America.

### Observed effects of nutritional interventions on risks for infection among ELTCRs

As described in Table 2, some nutrition-based interventions were observed and significantly influenced the risk of infection among ELTCRs, while others failed to do so.

**Table 2:** Intervention studies reporting a significant association between nutrition & infection risk.

Infection Related Outcome	Experimental Studies Reporting a Significant Effect of Nutrition-Based Interventions on Infection	Details on Association
Any Infection (Unspecified)	Björkman et al. (2012) [52]	**Protein:** Six months of supplementation with 20g/day whey protein significantly reduced the risk of developing any infection (not specific) (p = 0.009) [52]
Urinary Tract Infection	Handeland et al. (2014) [53]	**Black Chokeberry:** Three months of consuming 300 ml of Black Chokeberry juice a day significantly reduced the risk of UTI for the three-month period following intervention (p<0.05) [53].
Lymphocyte Parameters	Barnett et al. (2016) [55]	**Zinc**
		• 30 mg of zinc gluconate significantly increased T-cell counts (p = 0.03)
		• 30 mg of zinc gluconate, based on anti-CD3/CD28 assessment, significantly increased lymphocyte proliferation (p = 0.02)
	Zanini et al. (2017) [57]	**Meal Plan:** Lymphocyte counts significantly benefited from a meal plan that emphasized texture, nutrient density, and variety in dysphagic ELTCRs (ANOVA test for blood lymphocyte count changes over 12 months: F = 215.39, p<0.001) (Z-score = 19.63, p<0.001)
Acute Respiratory Infections	Ginde et al. (2017) [54]	**Vitamin D:** 100,000 IU vitamin D3/month, for 12 months, significantly reduced the incidence of both general (IRR: 0.60; 95% CI: 0.38–0.94, p = 0.02) and upper acute respiratory tract infections (IRR: 0.53; 95% CI: 0.31–0.90, p = 0.02) [54]
Skin and Soft Tissue Infections	Ginde et al. (2017) [54]	**Vitamin D:** 100,000 IU vitamin D3/month, for 12 months, significantly reduced the incidence of skin and soft tissue infections (IRR: 0.32; 95% CI: 0.13–0.80, p = 0.02) [54]

IRR: Incidence rate ratio.

CI: Confidence interval.

ANOVA: Analysis of variance.

#### Probiotics

None of the three probiotic-based experimental studies reported a significant benefit of supplementation for any infection related outcome [49–51]. A five-gram multi-strain probiotic (10^10^ colony forming unit, CFU) product, delivered two times a day, beginning at the onset of antibiotic therapy until one-week post completion, did not reduce the frequency of antibiotic usage/prescriptions throughout the follow-up period [49].

Compared to placebo, two bottles a day, for 176 days, of a fermented milk product containing *Lactobacillus casei Shirota* (6.5 x 10^9^ CFU) did not significantly reduce the number of days with respiratory infection symptoms (p = 0. 342), nor the number of participants presenting with respiratory symptoms (p = 0.325), based on univariate analysis [50]. Multiple logistic regression analysis revealed that *Lactobacillus casei Shirota* did not significantly alter the risk of developing a severe respiratory tract infection, compared to placebo (OR: 0.592; 95% CI: 0.335–1.049) (p = 0.073) [50]. Further, probiotic supplementation did not significantly influence the rates of seroconversion or seroprotection between groups in this cohort who received influenza vaccination at 21 days post-intervention initiation [50].

Six months of supplementation with two capsules a day of *Lactobacillus rhamnosus GG* (10 billion CFU/capsule) did not significantly reduce the risk of developing a viral respiratory infection (PCR-confirmed), compared to placebo (HR: 0.65; 95% CI: 0.32–1.31) [51]. There were no significant group differences for any of the secondary outcomes, including influenza-like illness, antimicrobial prescriptions, physician visits for respiratory illness, hospitalization for lower respiratory infections or pneumonia, rate of lower respiratory infections, or pneumonia [51].

In another vein, while it did not significantly affect an infection related outcome, supplementation with a multi-strain probiotic significantly reduced the number of antibiotic-associated diarrhea episodes (p = 0.022), possibly signalling an option to improve treatment tolerance and compliance [49].

#### Protein

Two intervention studies provided nursing home residents with supplemental protein, yielding mixed results [52, 56].

Twenty grams of whey protein, dissolved in 4.5 deciliter (dL) of juice, consumed in three equal portions a day for 6 months, did not significantly reduce risk of urinary tract infections, compared to control (juice alone) (p = 0.053) [52]. Over a 6 month follow-up, residents in the intervention arm experienced significantly fewer infections overall (not specified), compared to control (p = 0.009) [52].

At risk residents (BMI ≤ 24 kg/m^2^ & MNA score ≤ 24) consuming a warm soy drink product (9.5g protein, 250 kcal) once a day, over a 24-week period, did not significantly improve lymphocyte counts, compared to control (p>0.05) [56].

#### Black Chokeberry (Aronia melanocarpa)

One intervention trial, with crossover, providing residents with a Black Chokeberry product reported mixed results for different infection related outcomes [53]. Depending on the period of crossover, residents consumed either 300 ml of placebo or Black Chokeberry juice, once a day, for 3 months. Compared to the previous period, Black Chokeberry juice did not significantly alter the frequency of urinary tract infections, or any other infection (p>0.05) [53]. The number of urinary tract infections occurring over the three months following Black Chokeberry juice supplementation significantly decreased (p<0.05). However, no other infection rates were altered (p>0.05) [53].

#### Dedicated, needs-specific, meal plan

One single-arm, pre-post, clinical trial providing dysphagic (difficulty in swallowing) residents with a needs-specific meal plan, which was comprised of food options that included recommended amounts of protein, were textured, more nutrient dense, and varied compared to standard meals, reported positive results [57]. At the end of the 6-month intervention, compared to baseline values, 98.23% of residents achieved normal plasma lymphocyte values, with counts significantly benefiting from the meal plan [analysis of variance test for blood lymphocyte count changes over 12 months: F = 215.39, p<0.001] (Z-score = 19.63, p<0.001) [57].

#### Vitamin D

One intervention trial supplemented residents with a monthly high dose of vitamin D3 for 12 consecutive months, reporting overall positive results for most infection related outcomes [54]. Residents in the intervention arm received 100,000 international units (IU) of vitamin D3/month, whereas those in the control either received a placebo (if they were already supplementing with 400–1000 IU) or 12,000 IU vitamin D3/month if they were supplementing with <400 IU/day vitamin D. Throughout the study period, at all assessed time points, blood levels of vitamin D were significantly higher in the intervention group compared to control (p<0.001) [54]. The incidence of acute respiratory infections was significantly lower in the intervention arm compared to control (IRR: 0.60; 95% CI: 0.38–0.94, p = 0.02). However, the number of residents having at least one case did not significantly differ between groups (31% vs 46%, respectively) (p = 0.10) [54]. Compared to control, the incidence of specifically upper acute respiratory infection was significantly lower in the intervention arm (IRR: 0.53; 95% CI: 0.31–0.90, p = 0.02), with similar benefits observed for skin and soft tissue infections (IRR: 0.32; 95% CI: 0.13–0.80, p = 0.02) [54]. The incidence of urinary tract infection or any other infection, and hospitalizations for acute respiratory infections, did not differ between groups (p>0.05) [54].

#### Zinc

One small intervention trial, involving residents with low baseline serum zinc concentrations, reported mixed results of zinc gluconate supplementation effects on immunity [55]. Three months of supplementation with a multivitamin (providing ~50% of dietary reference intakes of essential vitamins and minerals) containing 30 mg of zinc gluconate significantly increased T-cell counts (p = 0.03) and improved lymphocyte proliferation (p = 0.02), compared to control (multivitamin with 5 mg of zinc) [55]. Notably, while the mean change in serum zinc levels were significantly higher in the intervention arm (p = 0.007), at the end of the study period, 42% residents still did not reach adequate concentrations.

### Association between nutritional deficits/malnutrition and risk of infection

Two observational, prospective cohort, studies were identified; one investigating the association between vitamin D deficiency and the risk of respiratory tract infections, hospitalizations, and any type of infection [26]. The other, focusing on the association between different nutritional assessment tools and development of any type of infection [58].

#### Vitamin D

The prevalence of vitamin D deficiency was observed to be significantly higher in residents experiencing respiratory tract infections than those with no deficiency [26]. A Kaplan-Meier curve calculation (probability over a period of time of surviving often including a parameter of interest, while accounting for participants departing) [60] demonstrated that the rate of respiratory tract infection-free survival was significantly lower among those with severe vitamin D deficiency (<10ng/mL) (p<0.001) [26]. Cox proportional hazard analysis indicated that vitamin D deficiency was a significant predictor of developing a respiratory tract infection [HR: 6.755 (95% CI: 3.693–12.358) (p<0.001)] [26].

#### Nutritional assessment tools

Neither the mini-nutritional assessment (MNA) tool, Nutritional Risk Screening 2002 (NRS) tool, or the Malnutrition Universal Screening Tool (MUST) at either the 6-month or 12-month time point was predictive of infection [58].

## Discussion

In this scoping review, the described literature landscape included interventions that significantly reduced the risk of infection, encompassing whey protein (any infection) [52], Black Chokeberry (urinary tract infection) [53], and vitamin D (acute respiratory tract infection, skin infection, and soft tissue infection) [54]. Both zinc and a dedicated meal-plan significantly improved lymphocyte parameters [55, 57]. Vitamin D deficiency was associated with the risk of developing respiratory tract infections [26].

Vitamin D is a well-documented modulator of both the innate and adaptive immune system with notable influence on antimicrobial peptide production, inflammatory cascade regulation, gene expression, and T-cell activity [61]. Clinically, vitamin D status is associated with the risk of developing infections, such as SARS-CoV-2 [62], acute respiratory tract infections [63, 64], urinary tract infections [65], and tuberculosis [66]. In this scoping review, a high dose (100,000 IU/month) of vitamin D3 supplementation within an elderly long-term care setting reduced the risk of developing both general and upper acute respiratory tract infections, as well as skin and soft tissue infections [54]. This dose translates to between 3226 and 3571 IU/day. While a culture of caution exists within the medical community for the use of large bolus doses of vitamin D causing hypervitaminosis toxicity, the totality of evidence indicates that interventions similar to that identified in this review [54] may be well tolerated [67–70]. This review further identified that the lack of correction of suboptimal vitamin D levels can result in a substantially higher respiratory infection burden among ELTCRs [26].

Dietary protein supplies necessary organic compounds, termed amino acids [71], which support vital components of the immune system, including T-cell and B-cell activation, lymphocyte proliferation, gene expression, and antibody production [72]. Protein-malnutrition among ELTCRs is common in Canada [29, 30]. As described by Björkman and colleagues, a supplement of twenty grams of whey protein per day reduced the risk of developing any infection among ELTCRs [52]. The use of whey protein provides a rich source of amino acids involved in immune system activity [73]. Thus, supplementation may be a viable dietary-based addition to infection prevention.

Black Chokeberry (*Aronia melanocarpa*) fruit is a rich source of bioactive phytochemicals, including anthocyanins, proanthocyanidins, flavonols, flavanols, and phenolic acids [74]. With a similar chemical profile as cranberry, which has been observed to reduce the risk of urinary tract infections [75, 76], our scoping review reports that Black Chokeberry juice reduced the risk of developing a urinary tract infection for up to three months post-intervention [53].

Lymphocytes, which include T-cells, B-cells, and natural killer cells are involved in essential immune system functions, such as antibody production, cell-mediated (direct) killing of virus-infected cells, and regulating immune responses [77]. Lymphopenia has been associated with an increased risk of infection-related hospitalizations and infection-related mortality for the general population [78], and more recently, associated with inferior outcomes among the elderly infected with SARS-CoV-2 [79]. Zinc, a trace mineral crucial for a normal functioning immune system [80], was identified in our scoping review as a possible intervention to increase both T-cell counts and lymphocyte proliferation [55]. So far, the majority of research with reference to zinc supplementation and infection prevention has been focused on children [81–83]. However, this review identified a possible new target population at the opposing end of the age spectrum. A meal plan facilitating consumption of a variety of textured, nutrient dense, foods also increased lymphocyte counts for dysphagic ELTCRs [57].

In relation to this current scoping review, age-related changes in the human body are associated with a decline in immune system functions, which affect both innate and adaptive immunity and the provision of nutritional interventions may help prevent infectious sequelae [84]. Diet is considered a major influence in the management to prevent the onset of infections [85]. Nutrient deficiencies may cause the immune system to decrease in defences against invading pathogens, as well as increase susceptibility to infection [86]. These deficiencies can be corrected through diets rich in, or supplements containing, essential vitamins and minerals. Infectious agents such as viruses, bacteria, protozoa and parasitic worms can interfere with factors, such as the availability of nutrients on specific components of the immune system related to the host nutritional state, further increasing susceptibility to infection [87, 88]. An example demonstrating the intricate relationship between the immune system and nutrition is exemplified by a component of the intestinal immune system known as the gut-associated lymphoid tissue (GALT) [88]. The GALT becomes impaired in the absence of adequate nutrition which in order to optimally prevent invasion of pathogens requires amino acids, glutamine, arginine; nucleotides; omega-3 fatty acids; and dietary fiber [88, 89]. Furthermore, the process of aging is related to changes in the composite of the gut microbiota that may increase infection among the elderly population [90]. Another example that affects the elderly population is seasonal influenza. Older adults with chronic medical conditions and /or immunological disorders are at greater risk for influenza infection [91]. Vitamin D has been observed to augment cytokine production and lymphocyte proliferation, improve pathogen clearance and possibly improve mucosal and systemic antibody responses when co-administered with influenza vaccine [91]. Thus, maintaining vitamin D levels among the elderly population through diet and supplements may decrease susceptibility to seasonal influenza [91]. In addition to the provision of a balanced diet, vitamins and minerals, other positive lifestyle factors, such as regular low intensity exercise and a routine with adequate hours of sleep will influence the immune system towards infection prevention while promoting health and wellness among an elderly population [92, 93].

### Limitations

Although effective in many ways it is noteworthy that the following limitations were encountered in this scoping review. The protocol used for this scoping review was not registered with PROSPERO and the study included only peer reviewed published articles, while grey literature was not included within the area of interest. This approach was taken to minimize articles which are not a product of peer-review processes. Additionally, the other challenge with grey literature is that many resources may not be retained on an online platform to facilitate searchable solutions. The exclusion of non-longitudinal studies such as cross-sectional studies were a result of the impact of time required for the effectiveness of the nutrition-based intervention or supplement. Our scoping research review describes an elderly cohort living in long-term care facilities that undergo surveillance over time to document the incidence of an outcome (a reduction in infection) among exposed (nutrition-based intervention) and non-exposed participants. The temporality of a prospective or longitudinal study affords the cohort exposure time (nutritional intervention) with each participant observed repeatedly. We believe the time factor in such longitudinal designs is appropriate for this research work as it affords an explanation of causality. Case reports were excluded as they did not fit the designation of a trial as well ranking of validity is lower compared to experimental or prospective cohort studies. This scoping review only included recent studies published within the past 10 years. While this increases the relevance and applicability to modern cohorts, it does not exhaustively describe the overarching research landscape for this topic beyond this time frame.

## Conclusion

Review of the current literature was effective in identifying the use of nutrition-based interventions for infection prevention among ELTCRs. In this scoping review, some nutrition-based interventions were observed and significantly influenced the risk of infection among ELTCRs. The nutritional interventions best poised for immediate rigorous clinical trial evaluation and subsequent implementation for ELTCRs are vitamin D (preventing deficiency/insufficiency), Black Chokeberry juice, zinc gluconate, whey protein, and varied and nutrient dense meal plans. Probiotics are unlikely to be of much value for infection prevention/control in this specific at-risk population. Overall, there is minimal research for nutrition-based interventions for infection prevention among ELTCRs, warranting more intervention studies, beginning with the ones identified in this review.

## Supporting information

S1 File Annotated bibliography. (PDF)

S1 Table PRISMA-ScR-checklist. (PDF)
