# Supplementary material for: Contemporary nutrition-based interventions to reduce risk of infection among elderly long-term care residents: A scoping review
Source: PLoS One. 2022 Aug 2;17(8):e0272513. doi: 10.1371/journal.pone.0272513 (PMC9345473; doi:10.1371/journal.pone.0272513)
Supplement: S1 File — (PDF) [file pone.0272513.s001.pdf]

Appendix 1: Annotated Bibliography

1. **Barnett JB**, Dao MC, Hamer DH, et al. Effect of zinc supplementation on serum zinc concentration and T cell proliferation in nursing home elderly: a randomized, double-blind, placebo-controlled trial. Am J Clin Nutr. 2016;103(3):942-951. doi:10.3945/ajcn.115.115188

    The randomized, double-blind, placebo-controlled trial by Barnett et al., published in 2016, explores the magnitude of effect that zinc gluconate (30 mg/day for three months) has on lymphocyte parameters in nursing home residents with low serum zinc (<70 µg/dL). Even though zinc serum levels significantly increased in the active arm compared to control, 42% of residents still did not reach adequate concentrations, all of whom had baseline values ≤ 60 µg/dL. The observed T-cell proliferation enhancement was determined to mainly be due to an increase in the total number of cells, rather than the capacity to proliferate. It is difficult to determine if the observed effects were only due to zinc, as the intervention also contained low amounts of other vitamins and minerals which may independently or synergistically alter immunity. Further, because of a small initial sample size ($n=31$) and a subsequent attrition rate of 19.4%, these effects cannot yet be generalised to other populations. The authors conclude that zinc gluconate enhances T-cell function primarily by increasing the number of functioning peripheral cells, and is both feasible and safe.

2. **Björkman MP**, Finne-Soveri H, Tilvis RS. Whey protein supplementation in nursing home residents. A randomized controlled trial. European Geriatric Medicine. 2012;3(3):161-166. doi:10.1016/j.eurger.2012.03.010.

    The randomized controlled trial by Björkman et al., published in 2012, investigated multiple outcomes, primarily anthropometric, of whey protein supplementation (20g/day) among 106 elderly nursing home residents over a six-month period. Two infection related secondary outcomes were explored in 93 evaluable participants; 1) decrease in the number of any infections at 180 days and 2) decrease in the number of urinary tract infections (UTIs) at 30 days, compared to baseline. Regarding any infection, the control group had one less infection compared to a decrease of nine in the intervention group ($p=0.009$), whereas no significant difference in the reduction of UTIs was observed ($p=0.053$). It is difficult to confidently determine if the reduction in infection rate (any type) was due specifically to whey protein, as it was mixed with juice which contains a mix of vitamins, minerals, and phytochemicals, while also providing calories. Further, all residents were encouraged, to varying degrees based on ability, to exercise using a stationary bicycle. The overall approach undertaken in this trial effectively increased body weight and skeletal muscle profiles of residents, ultimately reducing the need for physical assistance and increasing independence/wellbeing.

3. **Diekmann R**, Winning K, Uter W, et al. Screening for malnutrition among nursing home residents - a comparative analysis of the mini nutritional assessment, the nutritional risk screening, and the malnutrition universal screening tool. J Nutr Health Aging. 2013;17(4):326-331. doi:10.1007/s12603-012-0396-2

    The prospective cohort study by Diekmann et al., published in 2013, evaluated the predictive value of three validated nutritional assessment tools among 200 nursing home residents for several endpoints. Baseline assessment involved the Mini Nutritional Assessment (MNA), the Nutritional Risk Screening 2002 (NRS), and the Malnutrition Universal Screening Tool (MUST). While nutritional status, as determined by all three tools, was associated with risk of mortality, none showed an association between scores and risk of infection. As this cohort included participants with cognitive impairments, a group historically excluded from similar studies, the results of this observational investigation are more generalizable to a wider range of nursing home residents. It is difficult to quantity the level of bias that may have been introduced by the chosen data collection method (the MNA was only completed by nursing staff, whereas the NRS and MUST were completed by two of the study authors using medical records), thus results should be interpreted judiciously. The authors appropriately conclude that there is a substantial degree of heterogeneity of nutritional status scores between tools which is meaningful when making intervention decisions based on assessment instruments.

4. **Ginde AA**, Blatchford P, Breese K, et al. High-Dose Monthly Vitamin D for Prevention of Acute Respiratory Infection in Older Long-Term Care Residents: A Randomized Clinical Trial. J Am Geriatr Soc. 2017;65(3):496-503. doi:10.1111/jgs.14679

    The randomized, double-blind, placebo-controlled Phase II trial by Ginde et al., published in 2017, evaluated the effectiveness of high dose vitamin D3 (100,00 IU/month) for reducing the risk of acute respiratory infection (ARI) among 107 elderly long-term care residents. The high dose vitamin D3 intervention significantly outperformed the standard dose for ARI (any type) incidence, but not for hospitalisations or other infections. As with such a high dose of vitamin D, safety was also evaluated. Based on prespecified adverse events (AEs) of vitamin D (hypercalcemia, nephrolithiasis, or hypervitaminosis D), there were no significant differences between groups (no occurrences of any AE in either). The high dose vitamin D group experienced significantly more falls ($p<0.001$), however, authors determined that individual participants reporting multiple falls was likely inflating the difference. No increase in fractures was observed.

5. **Handeland M**, Grude N, Torp T, Slimestad R. Black chokeberry juice (Aronia melanocarpa) reduces incidences of urinary tract infection among nursing home residents in the long term--a pilot study. Nutr Res. 2014;34(6):518-525. doi:10.1016/j.nutres.2014.05.005

    The group-randomized, double-blind, placebo controlled, crossover trial by Handeland et al., published in 2014, explored the effects of Black Chokeberry (*Aronia melanocarpa*) juice (300 ml/day) on the incidence of UTIs among 236 elderly nursing home residents. The mean actual amount of consumed juice in one group was 156 ml and 89 ml in the

other, with the first experiencing a 55% reduced UTI incidence compared to 38% in the other, indicating a possible dose response relationship. In this cohort the intervention effect was observed during the preceding three months, indicating a delayed response. While the crossover design of this trial strengthens the confidence in the observed association, as the mean consumption rate was substantially below the intended intervention, feasibility should be considered as a possible barrier to external application. It should be noted that residents were receiving simultaneous prophylactic UTI treatments, including cranberry products and antibiotics. Authors conclude that, as this is the first study of Black Chokeberry intervention to reduce the incidence of UTIs, further studies are warranted to confirm the encouraging results reported in this pearly phase clinical trial.

6. **Kuwabara A**, Tsugawa N, Ao M, Ohta J, Tanaka K. Vitamin D deficiency as the risk of respiratory tract infections in the institutionalized elderly: A prospective 1-year cohort study. *Clin Nutr ESPEN*. 2020;40:309-313. doi:10.1016/j.clnesp.2020.08.012

   The prospective cohort study by Kuwabara et al., published in 2020, explored the association between 25(OH)D serum levels and the incidence of respiratory tract infections (RTIs) among 208 nursing home residents. With a median observation period of 354.2 days, 25(OH)D serum levels were associated with the incidence of RTIs, with deficiency also being more prevalent among those developing disease. Furthermore, iPTH, a biomarker inversely associated with vitamin D levels, was higher among those developing RTIs, reinforcing the evidence of an association between 25(OH)D deficiency and risk of illness. As authors report that detailed medical records could not be obtained, in addition to smoking history, there is possible covert confounding that may have biased results. It is noteworthy that a propensity score matching approach was applied in order offset potential bias and improve the accuracy of the association between exposure (vitamin D levels) and outcome (RTIs).

7. **Lee LC**, Tsai AC, Wang JY, Hurng BS, Hsu HC, Tsai HJ. Need-based intervention is an effective strategy for improving the nutritional status of older people living in a nursing home: a randomized controlled trial. Int J Nurs Stud. 2013;50(12):1580-1588. doi:10.1016/j.ijnurstu.2013.04.004

   The randomized, double-blind, non-placebo-controlled trial by Lee et al., published in 2013, compared standard care and regular meals to the addition of a soy protein based warm drink preparation (9.5g protein, 250 kcal) among 92 nursing home residents. If a participant randomized to the intervention group was determined to be undernourished (MNA score ≤ 24 and BMI ≤ 24 kg/m^2) they received the additional need-based intervention. The only immune related outcome evaluated, lymphocyte count, did not significantly improve over the 24-week intervention. At baseline, of the 43 assigned to the intervention arm, 30 initially met the criteria for being malnourished, and an additional eight after the first four weeks, signalling a high prevalence of undernourished elderly residents. It is unclear how this intervention would alter nutritional status and biochemical markers of immune status (e.g., lymphocyte count) in more decompensated patients as this cohort involved residents that were all non-bed-ridden, were able to self-

feed and did not suffer from cognitive deficits. Authors conclude that a need-based intervention including a soy protein meal improved nutritional status, based on measures such as BMI, however, it does not appear effective for improving lymphocyte count. As the incidence of developing an infection was not included as an outcome, it is not possible to comment on the clinical effectiveness of this intervention.

8. **Van Puyenbroeck K**, Hens N, Coenen S, et al. Efficacy of daily intake of Lactobacillus casei Shirota on respiratory symptoms and influenza vaccination immune response: a randomized, double-blind, placebo-controlled trial in healthy elderly nursing home residents. Am J Clin Nutr. 2012;95(5):1165-1171. doi:10.3945/ajcn.111.026831

   The randomized, double-blind, placebo-controlled trial by Van Puyenbroeck et al., published in 2012, explored the effectiveness of a single strain probiotic (*Lactobacillus casei* Shirota) product on respiratory tract infection incidence and response to influenza immunization in 737 nursing home residents. In this adequately powered trial, daily supplementation with 1.3 x 10^{10} live *Lactobacillus casei* Shirota cells for 176 days did not influence any respiratory tract infection endpoints, nor the immune response of influenza vaccination (administered 21 days after intervention initiation). A null effect was observed based on both univariate and multivariate analysis. A high attrition rate (183 dropouts) was reported; however, adverse events recorded as the reason for leaving the study were similar between groups, including gastrointestinal symptoms (24% for controls vs. 26% for the active arm). Authors conclude that this specific probiotic intervention does not influence respiratory tract infection incidence, nor immune response to influenza vaccination, for this specific population.

9. **van Wietmarschen HA**, Busch M, van Oostveen A, Pot G, Jong MC. Probiotics use for antibiotic-associated diarrhea: a pragmatic participatory evaluation in nursing homes. BMC Gastroenterol. 2020;20(1):151. Published 2020 May 13. doi:10.1186/s12876-020-01297-w

   The pragmatic participatory evaluation trial by van Wietmarschen et al., published in 2020, primarily looked at the use probiotics to manage antibiotic-associated diarrhea, including some relevant infection related endpoints in 93 nursing home residents. The multi-strain probiotic (10^{10} colony forming unit) product contained *Bifidobacterium bifidum W23, Bifibacterium longum W51, Enterococcus faecium W54, Lactobacillus acidophilus W37 and W55, Lactobacillus paracasei W20, Lactobacillus plantarum W62, Lactoba- cillus rhamnosus W71, and Lactobacillus salivarius W24*. Residents prescribed amoxicillin/clavulanic acid or ciprofloxacin were prescribed the probiotic product, two times daily, until one week after completion of the antibiotic regimen. This administration scheme did not alter the number of antibiotic requirements between those receiving probiotics or not. It did, however, significantly reduce the number of antibiotic-associated diarrhea episodes experienced by residents, indicating an approach to improving tolerance, and possibly compliance. As this study was not designed to tract nor influence the incidence and/or immune status of elderly residents, similar probiotic interventions with differing administration schemes and evaluations are warranted. Authors concluded that this intervention is promising for the management of diarrhea side-effects often experienced by residents receiving antibiotics.

10. **Wang B**, Hylwka T, Smieja M, Surrette M, Bowdish DME, Loeb M. Probiotics to Prevent Respiratory Infections in Nursing Homes: A Pilot Randomized Controlled Trial. J Am Geriatr Soc. 2018;66(7):1346-1352. doi:10.1111/jgs.15396

    The randomized, double-blind, placebo-controlled pilot trial by Wang et al., published in 2018, explored the effectiveness of daily supplementation with a single strain probiotic (20 billion cfu/day of *L. rhamnosus GG*) for six months on the incidence of laboratory-confirmed respiratory tract infections (viral origin). Primary outcome viral infections (confirmed by polymerase chain reaction) included influenza A & B, entero-rhino virus, parainfluenza 1,2, and 3, metapneumovirus, and respiratory syncytial virus (RSV). There were no significant differences for any of the tested viral respiratory infections between groups, nor any differences regarding secondary outcomes, including, symptomatic respiratory infections, pneumonia related hospitalization or emergency departments visits for respiratory infections. None of the adverse events were reported to be due to the study intervention. A future larger trial is warranted, as authors report that their study was underpowered, resulting in a non-significant test statistic result, even though a 35% risk reduction was observed in the intervention arm compared to placebo.

11. **Zanini M**, Bagnasco A, Catania G, et al. A Dedicated Nutritional Care Program (NUTRICARE) to reduce malnutrition in institutionalised dysphagic older people: A quasi-experimental study. J Clin Nurs. 2017;26(23-24):4446-4455. doi:10.1111/jocn.13774

    The uncontrolled, pre-post, single-arm clinical trial by Zanini et al., published in 2017, investigated the effects of introducing a meal program to 479 dysphagic nursing home residents that emphasized food texture, variety, and nutrient density. Compared to baseline, lymphocyte parameters normalized in 98.23% of residents. It is difficult to predict if a similar meal plan program would yield similar results in other nursing home residents, as participants in this study all suffered from dysphagia and may represent a sub-population with unique responses to nutritional intervention.
